# Colorectal Carcinoma, Cyclooxygenases, and COX Inhibitors

**DOI:** 10.7759/cureus.28579

**Published:** 2022-08-30

**Authors:** Vinutna Ganduri, Kruthiga Rajasekaran, Shrimahitha Duraiyarasan, Mayowa A Adefuye, Nisha Manjunatha

**Affiliations:** 1 Research, Bhaskar Medical College, Hyderabad, IND; 2 Research, Rajah Muthiah Medical College & Hospital, Chidambaram, IND; 3 Research, K.A.P. Viswanatham Government Medical College, Tiruchirappalli, IND; 4 Research, University of Ibadan College of Medicine, Ibadan, NGA; 5 Research, Our Lady of Fatima University College of Medicine, Valenzuela, PHL

**Keywords:** gastrointestinal neoplasms, chemoprevention, celecoxib, aspirin, selective cox-2 inhibitors, (nsaid) non-steroidal anti-inflammatory drugs, cyclooxygenase inhibitors, cyclooxygenases, (crc) colorectal carcinoma

## Abstract

Colorectal carcinoma (CRC) is the most common of gastrointestinal cancers, the majority presenting with sporadic occurrence compared to the less frequently inherited syndromes. The increasing incidence, decreasing gender and age disparities, and the prevalent risk factors are concerning. The malignancy arising from benign precursor polyps transforms slowly over time. The adenoma variant polyps reported a marked upregulation of cyclooxygenases (COX), significantly COX-2 isoform, influenced by various determinants such as genetics, pathology, histology, and site of the carcinoma. These COX enzymes are responsible for prostaglandin synthesis and the consequent cascade of cell inflammation and proliferation. Therefore, COX inhibition by non-steroidal anti-inflammatory drugs (NSAIDs) targeted against both the isoforms COX-1 and COX-2 have been studied for decades in anticipation of preventing the occurrence of colorectal carcinoma in high-risk populations. This article has collated and highlighted the overexpression of COX enzymes by the adenomatous polyps and provides corroborating evidence from multiple studies in favor of COX inhibition by NSAIDs. Aspirin and Sulindac were two drugs to be initially proven to halt the progression and cause regression of the polyps. Celecoxib, a selective COX-2 inhibitor besides NSAIDs, was also used in experimental studies.

## Introduction and background

Colon cancer is the world's fourth most common cancer while rectal cancer is in the eighth position, according to GLOBOCAN 2018 data [1]. Colorectal carcinoma (CRC) accounts for 11% of all cancer diagnoses worldwide, making them the third most common type of cancer [1]. CRC is more frequent in men than women and is three to four times more common in developed countries than in developing nations [2]. In recent decades, the gender gap between older and younger adults has narrowed [3]. According to researchers, right-sided (proximal) colon cancer is more aggressive than left-sided (distal) colon cancer [4], and proximal colon cancer patients are more likely to be females than males [5]. The "Western lifestyle" is linked to an increased risk of colon carcinoma due to established risk factors such as red meat, alcohol intake, sedentary lifestyle, and obesity. Inflammatory bowel disease (IBD) is a significant risk factor for colon cancer and should therefore be monitored closely [6]. About 75% of CRC cases are sporadic without apparent evidence of inheritance, and the remaining 20-30% show familial clustering of genes, family history, and shared risk factors [6]. Familial adenomatous polyposis (FAP) and hereditary non-polyposis colorectal carcinoma (HNPCC) are two hereditary syndromes that have been associated with the development of CRC [6]. Colorectal malignancies develop from their benign precursors via two major pathways of the adenoma-carcinoma sequence or the serrated neoplasia pathway. As genetic changes accumulate, these benign polyps, in each case, slowly transform into cancer [7-9]. CRC presents clinically with rectal bleeding, change in bowel habits, and abdominal pain, which are more significant with cancer's distal location than proximal or right-sided cancer [10]. The diagnosis is made by visualizing the polyps through a colonoscopy and histological examination after a biopsy [11]. Colon cancer is treated based on staging, recurrence rate, and survival rates; surgical resection and chemotherapy are the options [12].

The use of non-steroidal anti-inflammatory drugs (NSAIDs) due to their inhibition of cyclooxygenase (COX) enzymes has been extensively researched in the hope of possible prevention of CRC growth and development [13]. Several studies published have reported a considerable decrease in CRC risk in those individuals who take aspirin regularly [14,15]. Although the role of NSAIDs in the risk reduction of CRC has not been completely understood, the involvement of arachidonic acid metabolites has been suggested in various stages of carcinogenesis [13]. This article aims to outline COX-2 expression by adenomatous polyps and discuss the role and mechanism of NSAIDs and selective COX-2 inhibitors in preventing the increased risk of CRC.

## Review

Pathogenesis of colorectal carcinoma

CRC development is an orderly process involving three phases of initiation, promotion, and progression through alterations in several genes, including oncogenes, tumor suppressor genes, and mutator genes [16,17]. These tumors are either sporadic, i.e., "de novo," or are hereditary with an autosomal dominant (AD) mode of inheritance [18,19]. The large intestine is characterized by specialized glands called crypts, constituting both columnar and mucinous cells, approximately 40-60 cells deep. The cells from the proliferative zone in the lower portions of the crypt migrate to the upper parts and are subsequently expelled from the mucosal surface [20]. Adenocarcinomas originate from these colonic epithelial cells. Initially, in carcinogenesis, the colonic epithelial cells undergo continuous replication. Subsequently, the proliferative zone expands, resulting in the distribution of the S-phase cells throughout the length of the gland. This generalized and disordered cell replication thus results in the ordered sequence of normal "mucosa- adenoma-carcinoma" upon accumulating genetic alterations at each phase [21,22]. The common genetic alterations include functional or mutational inactivation of genes involved in DNA mismatch repair (MMR genes) and abnormal DNA methylation besides the inactivation of tumor suppressor genes and the activation of oncogenes [22]. This process involves aberrant crypt foci (ACF), containing intestinal crypts with nuclear atypia and dysplasia. ACF is often presumed to be the precursor of adenomas and carcinoma [23]. The adenoma-carcinoma sequence explains the development of CRC from pre-existing adenomas, which are well-demarcated dysplastic epithelial tissue classified into various histological variants [24]. In a study by Vogelstein et al., 172 colorectal tumors spanning multiple phases of neoplastic growth along the adenoma-carcinoma sequence were examined for ras gene mutations and allelic deletions at chromosomes 5, 17, and 18. Thereby, a model was put forth proposing the accumulation of changes affecting at least the k-ras dominant oncogene and other tumor suppressor genes (inactivated by allelic loss) in the progression of CRC from normal mucosa to infiltrating carcinoma [22]. The initiating event in most sporadic carcinogenesis was proposed to be the loss of heterozygosity (LOH) at chromosome 5q and the genetic abnormalities at the adenomatous polyposis coli (APC) locus. This model was based on the detailed microallelotyping study by Boland et al. in which colorectal neoplasms were analyzed to determine the sequence and timing of the genetic alterations in tumorigenesis [25]. The main molecular events of sporadic CRC are the somatic inactivation (loss or mutation) of APC, resulting in disorganized mucosal cell proliferation, adhesion, and migration along the crypt axis [25,26]. These mutations result in abnormal adhesion and migration of cells, as the APC gene act as the "gatekeeper" of epithelial cell replication and its standard conditioning [27,28].

FAP is a hereditary syndrome with AD mode of inheritance of a constitutional mutation in one of the alleles of the APC gene [29]. A "second hit" phenomenon or the inactivation of the wild-type allele in the colonic mucosal cells is responsible for the development of polyps in the second decade of life, resulting in the gradual development of adenomas in the large bowel [30,31]. On complete inactivation of the pre-existing germline mutated APC gene, hundreds and thousands of polyps grow and present with a high probability of evolving into carcinoma. These polyps grow in size, become dysplastic, and eventually lead to infiltrating lesions upon accumulating genetic alterations in a much similar process as sporadic cases. Usually, these lesions begin from the recto-sigmoid region [32].

HNPCC (or Lynch syndrome) is a hereditary syndrome characterized by early neoplasia localized predominantly to the proximal colon (caecum to splenic flexure), synchronous and metachronous colorectal neoplasms, and frequently associated with tumors of other organs, in particular, endometrium, urinary tract, small bowel and ovary [33]. A constitutional mutation in one of the several MMR genes is inherited in AD mode, resulting in genomic instability at the microsatellite loci (microsatellite instability or MSI pathway). Though the function of the microsatellites is not clear, they are helpful in linkage studies due to their polymorphism [34,35]. Polyps in HNPCC are usually larger than sporadic polyps, often showcasing aggressive lesions (villous pattern and high-grade dysplasia), with a tendency to appear at an early age [36-38].

Ulcerative and Crohn's colitis are IBDs characterized by repetitive cycles of acute inflammation and mucosal regeneration [39]. Inflammation generates free radicals and other metabolites, which may induce specific changes promoting carcinogenesis. These changes include oxidative DNA damage with base substitution, breaks in DNA, sister chromatid exchanges, and several other mutations in cancer-related genes. The increased regeneration and replication may further increase the susceptibility of target cells to probable genetic alterations and cancer development [40].

What are cyclooxygenases?

Cyclooxygenases (COX-1 and COX-2) are enzymes that catalyze the rate-limiting step of prostaglandin synthesis [41]. COX-2 is an enzyme involved in regulating inflammation, cell proliferation, and angiogenesis via the synthesis of prostaglandins and thromboxanes from arachidonic acid. It is present at very low levels in the tissues. It is induced by stimuli such as interleukin-1 cytokine, growth factors, such as epidermal growth factor, transforming growth factor-beta, tumor necrosis factor-alpha, oncogenes ras and scr, hypoxia, benzo[a]pyrene, ultraviolet light [42,43]. The prostaglandin E2 (PGE2), a COX-2 metabolite, stimulates bcl-2 that inhibits the process of apoptosis, thereby inducing interleukin-6 (IL-6) production and enhancing the synthesis of haptoglobin. These mediators are responsible for the development and progression of the neoplasms; that is, PGE2 is responsible for tumor metastases, IL-6 for tumor invasion, and haptoglobin for implantation and angiogenesis [43,44].

COX-2 overexpression in CRC

The theory of COX-2 overexpression and the relative quantification of this enzyme has been reported in various premalignant and malignant lesions of epithelial origin; the gastrointestinal tract being the topic of the discussion [42,45]. It is reported in approximately 70-80% of esophageal, gastric, and colorectal carcinomas [46]. Apart from cancer cells, COX-2 was markedly expressed in inflammatory cells, vascular endothelium, and fibroblasts of the neoplastic tissues compared with non-lesional and normal colon tissues [45]. The frequency of COX-2 overexpression is noted in about 50% of adenomas and 80% of carcinomas, concluding that it is a frequent but not universal phenomenon [47]. Hennie MJ Roelofs et al. quantified the COX-2 mRNA levels by qPCR and normalized it with respect to tissue weight. The immunoreactive COX-2 mRNA was overexpressed in 80% of the tissue, whereas the mRNA levels of other housekeeping genes B2M and GADPH were expressed in 70% and 40% of the colorectal neoplastic tissue, compared to the normal neighboring colorectal mucosa, respectively [48]. Negi et al. examined the clinicopathological correlation of COX-2 levels in CRC patients. This study concluded that CRC tissues, compared to their adjacent normal tissues, expressed a significantly higher (p<0.05) relative quantity of COX-2 mRNA. In establishing a close association, it proposed the potential role of COX-2 as a biomarker in CRC diagnosis [49]. A similar study by Buskens et al. quantified COX-2 expression in esophageal adenocarcinoma arising from Barrett's esophagus (a product of chronic inflammation) in tumors resected from 145 patients undergoing curative surgery. These resected tumors were stained immunohistochemically with COX-2-specific anti-human monoclonal antibodies. About 79% of the carcinomas expressed moderate to solid immunoreactivity, indicating high COX-2 expression, while 21% showed negative to weak immunoreactivity. This relative COX-2 overexpression was also found to be correlated to distant metastases (p=0.02), local recurrences (0.05), and reduced survival rates (0.002), as evident among the above two groups [50]. The association between the clinicopathologic features and COX-2 expression was analyzed, and thus its prognostic significance using the student's t-test (continuous data) and the X2 test (categorical data) (Table 1) [50].

**Table 1:** The association between clinicopathologic features and COX-2 expression

Tumor characteristics.		COX-2	expression.	
		Low (n=30)	High (n=115)	P value
Depth of invasion.	T1 (45)	11	34	0.7
	T2 (17)	4	13	
	T3 (83)	15	68	
Lymph node involvement.	N0 (67)	18	49	0.09
	N1 (78)	12	66	
Distant metastases.	M0 (117)	26	91	0.6
	M1 (28)	4	24	
Tumor stage.	I (32)	7	25	0.7
	IIa (33)	9	24	
	IIb (16)	4	12	
	III (36)	6	30	

The histology, genetics, epigenetics, and the tumor's location all play a significant role in overexpression. Signet-ring cell carcinoma, MSI phenotype of sporadic colorectal carcinomas, and colon (proximal colon tumors frequently are MSI-positive tumors) compared to rectal carcinoma are all associated with remarkably lesser COX-2 expression [51,52]. Sporadic forms and almost half of the MMR-deficient CRCs do not show COX-2 overexpression because of the gene silencing due to hypermethylation of the COX-2 5’-CpG island [53].

Therefore, these findings implicate the lack of response to NSAIDs in sporadic tumors compared to its potential chemoprevention in HNPCC. Specific subgroups of CRCs may benefit from COX-2 targeted therapy. It is suggested that the treatment should be aimed at CRC prevention rather than its suppression after progression, especially in colorectal neoplasia, where marked expression is found [54,55]. All these studies confirm COX-2 upregulation in sub-epithelial tissue before its expression in epithelial tissue as cancer progresses, thus establishing the role of COX-2 in the early stages of CRC oncogenesis [55].

COX-2 inhibition and role of NSAIDs: COX as a potential molecular target in the prevention of colon carcinogenesis

COX inhibitors prevent the synthesis of arachidonic acid from prostaglandins, modulate tumor growth by altering stem cell gene expression, cause hypermethylation of the genes involved in cell proliferation and differentiation, suppress the promotion of angiogenesis, and inhibit WNT/CTNNB1 signaling, and thus promote apoptotic cell death [56-60]. The two isoenzymes COX-1 and COX-2, the targets of NSAIDs, also show selective inhibition by analgesic/antipyretic drugs such as acetaminophen, phenacetin, antipyrine, and dipyrone [41]. NSAIDs, for example, include aspirin, ibuprofen, nimesulide, and sulindac acid, all acting via different signaling pathways. For example, ibuprofen and indomethacin reversibly inhibit COX-2 by binding to its active site; aspirin irreversibly inhibits COX-2 by acetylating its active site [61]. Celecoxib potently suppresses prostaglandin-induced angiogenesis by COX-2 inhibition [62]. An experimental study in 1980 investigated the effect of indomethacin on chemically induced adenocarcinoma in rats. The rats were given intraperitoneal methylazoxymethanol acetate once every six weeks for 25 weeks to induce large bowel carcinoma chemically. These rats were then divided into five groups to receive intrarectal instillation of distilled water with the following drugs - indomethacin, hydrocortisone, PS-K, vehicle alone, and no treatment respectively, daily for three weeks. Groups 1 and 2 that received indomethacin and hydrocortisone, respectively, demonstrated a significantly lower number of tumors per tumor-bearing rat and suppression of colon carcinogenesis (progression of microscopic lesions into large polyps) in rats by indomethacin, an NSAID, thus paving the way for many studies since chemically-induced gastrointestinal tumors in rodents are similar to those in humans, both pathologically and immunologically [63]. In 1988, a case-control study of a large population conducted to assess the risk of colorectal carcinoma in relation to several chronic diseases, operations, and medications demonstrated a statistically significant difference among the cases with regard to the use of aspirin and aspirin-containing compounds. This epidemiological and clinicopathological study thus found a reduction in colon cancer incidence in regular aspirin users [64].

Before diving deep, let us establish three findings or evidence for NSAID chemoprevention. First, through selective inhibition, some NSAIDs induce the regression of adenomatous polyposis in FAP cases. Giardielo FM et al. conducted a study to assess the efficacy of sulindac at a dose of 150 mg twice daily in patients with FAP. A cohort of 22 patients was divided into two groups of those who had not undergone colectomy (including 18 patients) and those who had a subtotal colectomy with ileorectal anastomosis (including 4 patients), and each group was randomized separately. The endoscopic evaluation of the polyp number and size was done every three months for one year. The effect of sulindac therapy was evident after only three months, with a more significant reduction in the polyp burden (number and size) before the sixth rather than the ninth month [65]. Second, by reducing the number and size of colon adenomas, NSAIDs induce early disruption of the adenoma-carcinoma sequence and thereby suppress the subsequent carcinogenesis at the adenoma stage, as demonstrated in both familial and sporadic adenomas [65,66]. Third, multiple studies (Thun et al., Marnett et al., Giovannucci et al., et al.) showed people taking aspirin or NSAIDs regularly reported a 40-50% lower risk of developing CRC and reduced mortality [15,67-70].

In 1991, the most extensive study of NSAIDs chemoprevention on colon cancer was conducted by following over 600,000 individuals for colon cancer death and aspirin use. The rate of colon cancer death was inversely related to aspirin use. The relative risk of fatal colon cancer was 0.60 in men and 0.58 in women who used aspirin 16 or more times per month [15]. Randomized controlled trials (RCT) to establish the effectiveness of the COX inhibitors (including aspirin, sulindac, and celecoxib) were conducted through 2003. Aspirin thus showed a reduced risk of recurrent sporadic adenomas. These studies (combined evidence from 3 RCTs) also put forth evidence of adenoma regression in FAP with short-term use of NSAIDs (one to three years) [71]. Matsuhashi et al. concluded that sulindac can cause sporadic colorectal adenomatous polyps regression. According to experimental research, sulindac shrunk (greater than 40% decrease in diameter) in 13 out of 20 sporadic adenomatous polyps in 15 patients with a 300 mg/day regimen for four months. The patients with responsive polyps were significantly (p < 0.05) older (mean age of 59.8 years) than those with non-responsive polyps (mean age of 52 years); though not statistically significant (p = 0.12), female patients were slightly more resistant than males to the given treatment [72]. Likewise, sulindac also induced regression of polyps in FAP. Waddel WR et al. published case reports of a family with Gardner's syndrome. Out of four patients, three of them who underwent subtotal colectomy with ileoproctostomy had residual polyps in the rectal mucosa. On treatment with sulindac, these polyps almost wholly disappeared. The fourth patient with diffuse polyposis of intact/ unresected colon only presented with three small mucosal polyps after a year of sulindac therapy [73,74].

Although most NSAIDs suppress both the isoforms, the selective COX-2 inhibitors, such as celecoxib, which is 300 times more active against COX-2 than it is against COX-1, showed clinical chemoprevention besides pre-clinical anti-cancer activity [75,76]. They have been found to suppress experimental colon carcinogenesis in familial polyposis and sporadic adenomas [77-79]. In an experimental study, 77 patients with intact colorectum or partially resected colon and five or more familial adenomatous polyps about 2 mm in diameter were randomly assigned to placebo or celecoxib (100 or 400 mg twice daily) therapy groups for six months. Endoscopy was done at the beginning and as a follow-up at the end of the study. It was concluded that a regimen of 400 mg of celecoxib was associated with significant regression of the adenomas compared to the 100 mg regimen. Celecoxib (400 mg regimen) therapy showed a 28% reduction in the mean number of polyps and a 30.7% reduction in polyp burden/diameter, whereas the placebo group showed a reduction of 4.5% in the mean polyp number and 4.9% in the polyp burden [80]. A similar RCT by Arber N et al., with subjects chosen from across the world, was evaluated through cecal intubation and photographic documentation for any changes from the baseline polyp size upon selective COX-2 inhibition therapy. A daily dose of 400 mg celecoxib showed a reduced burden of adenoma and their size at the first follow-up colonoscopy at the end of 1 year and the second follow-up in year 3. It also reported a lower cumulative detection rate of advanced adenomas [81].

Based on a study by O'Brien et al. [82], which identified CD133 as a marker for cancer stem cells (CSC) in CRC, Yanhong Deng et al. found that membrane CD133 expression in HT29 (COX-2 positive) and DLD1 (COX-2 negative), two human colon cancer cell lines were downregulated on celecoxib treatment, which was confirmed by Western blotting and qPCR. Though this effect of celecoxib is COX-2-independent and is probably unique among COX inhibitors is worth mentioning. It was noted that this downregulation of CD133 expression was through inhibition of the Wnt signaling pathway, and it was time- and dose-dependent. The Wnt signaling pathway, activated upon APC disruption, controls the cell differentiation and renewal process frequently involved in tumorigenesis. It was reported that celecoxib increased the proportion of the genes involved in differentiation, thus reducing the genes involved in multiplying stem cells [83,84]. The Wnt signaling pathway is abnormally activated upon the accumulation of β-catenin mutations. This damages the colonic crypt structure, leading to increased CD133 expression and inducing the development of intraepithelial neoplasia by high-grade adenomas, accounting for about 80% of sporadic CRC [85,86]. Henceforth, CD133 could be a useful biomarker in identifying patients who might benefit from celecoxib chemoprevention since it is no more expressed in cell differentiation [87,88].

An observational study by Ng K et al. regarding the efficacy of aspirin and COX-2 inhibitor in stage III CRC patients enrolled in an adjuvant chemotherapy trial reported improved outcomes (Table 2).

**Table 2:** The efficacy of aspirin and COX-2 inhibitor in stage III CRC patients enrolled in an adjuvant chemotherapy trial CRC: colorectal carcinoma

		Multivariable-Adjusted Hazard risk (95% CI)		
		Recurrence-free survival.	Disease-free survival.	Overall survival.
Aspirin (consistently during and after completion of adjuvant chemotherapy) in 799 patients	All	0.51 (0.28 to 0.95)	0.68 (0.42 to 1.11)	0.63 (0.35 to 1.12)
	Secondary analysis censored at 5 years		0.61 (0.36 to 1.04)	0.48 (0.23 to 0.99)
COX-2 inhibitor (Celecoxib / Rofecoxib after completion of chemotherapy) in 843 patients	All	0.53 (0.27 to 1.04)	0.60 (0.33 to 1.08)	0.50 (0.23 to 1.07)
	Secondary analysis censored at 5 years		0.47 (0.24 to 0.91)	0.26 (0.08 to 0.81)

A statistically significant association between aspirin/COX-2 inhibitor use and reduced recurrence and mortality in stage III colon cancer patients was thus concluded [61].

## Conclusions

Colorectal carcinoma and non-steroidal anti-inflammatory drugs are often studied in tandem. NSAIDs and exceptionally COX-2 inhibitors were extensively explored and investigated in preventing CRC. This article reviewed the pathogenesis of CRC in association with the variability in COX-2 expression to provide a clearer insight into the potential adenocarcinomas that could benefit from targeted therapy. Multiple studies mentioned above and many others elucidated the marked expression of cyclooxygenases and successfully established a favorable ground for further and future research in COX inhibition and plausible CRC chemoprevention. COX inhibition has provided statistically significant results in reducing the increased risk of familial colorectal carcinoma compared to sporadic forms. In conclusion, the clinical implication of this review article is to provide relevant evidence and highlight the results achieved thus far and, therefore, lay out a basis for advancements in the role and use of NSAIDs in reducing the progression of colorectal adenocarcinomas. We recommend future studies in this aspect of medicine to uncover future therapeutic and prognostic components for colon cancer.
